# Foramen lacerum impingement of trigeminal nerve root as a rodent model for trigeminal neuralgia

**DOI:** 10.1172/jci.insight.168046

**Published:** 2023-06-08

**Authors:** Weihua Ding, Liuyue Yang, Qian Chen, Kun Hu, Yan Liu, Eric Bao, Changning Wang, Jianren Mao, Shiqian Shen

**Affiliations:** 1 Department of Anesthesia, Critical Care and Pain Medicine, Massachusetts General Hospital, Harvard Medical School, Boston, Massachusetts, USA.; 2 McGovern Institute for Brain Research and Department of Brain and Cognitive Sciences, Massachusetts Institute of Technology, Cambridge, Massachusetts, USA.; 3 Department of Pathology, Tuft University School of Medicine, Boston, Massachusetts, USA.; 4 Martinos Center for Biomedical Imaging, Department of Radiology, Massachusetts General Hospital, Boston, Massachusetts, USA.; 5 Brooks School, North Andover, Massachusetts, USA.

**Keywords:** Neuroscience, Pain

## Abstract

Trigeminal neuralgia (TN) is a classic neuralgic pain condition with distinct clinical characteristics. Modeling TN in rodents is challenging. Recently, we found that a foramen in the rodent skull base, the foramen lacerum, provides direct access to the trigeminal nerve root. Using this access, we developed a foramen lacerum impingement of trigeminal nerve root (FLIT) model and observed distinct pain-like behaviors in rodents, including paroxysmal asymmetric facial grimaces, head tilt when eating, avoidance of solid chow, and lack of wood chewing. The FLIT model recapitulated key clinical features of TN, including lancinating pain–like behavior and dental pain–like behavior. Importantly, when compared with a trigeminal neuropathic pain model (infraorbital nerve chronic constriction injury [IoN-CCI]), the FLIT model was associated with significantly higher numbers of *c-Fos*–positive cells in the primary somatosensory cortex (S1), unraveling robust cortical activation in the FLIT model. On intravital 2-photon calcium imaging, synchronized S1 neural dynamics were present in the FLIT but not the IoN-CCI model, revealing differential implication of cortical activation in different pain models. Taken together, our results indicate that FLIT is a clinically relevant rodent model of TN that could facilitate pain research and therapeutics development.

## Graphical abstract

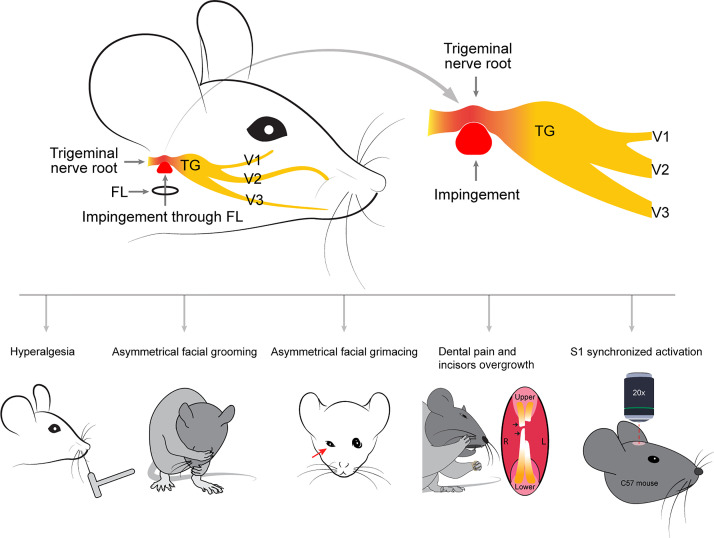

## Introduction

Trigeminal neuralgia (TN) is one of the most painful conditions in humans. As such, it has been called “the suicide disease” in mass media (1, 2). It is often caused by impingement or demyelination of the trigeminal nerve root, particularly at its entry zone. TN often presents with highly characteristic paroxysmal pain attacks, facial tics, and well-defined trigger zones (3–5). With these unique features, TN is a distinct diagnosis that is different from trigeminal neuropathic pain, which is any neuropathic pain in the trigeminal territory (6–8).

Seminal work from Yeomans et al. and Luo et al. used stereotaxic access from the brain and from the infraorbital fissure, respectively, to compress the trigeminal nerve root (9, 10). In both cases, significant mechanical allodynia was observed. Importantly, Yeomans et al. reported that bodies of rats shook when rats were eating, presumably due to triggered dental pain. This phenomenon was consistent with commonly reported oral trigger zones in patients with TN. Inspired by previous work, we sought to develop a clinically relevant rodent model of TN to recapitulate its key clinical features. Recently, we reported the foramen lacerum impingement of trigeminal nerve root (FLIT) model (11). Herein, we described the skull base anatomy underlying the FLIT model, demonstrated the tissue injury associated with the FLIT surgery, optimized surgical technique across different species, and characterized behavioral features of this model. Additionally, we unraveled unique cortical activation patterns in TN by using this model.

## Results

### Anatomy of the foramen lacerum.

**Figure 1:**
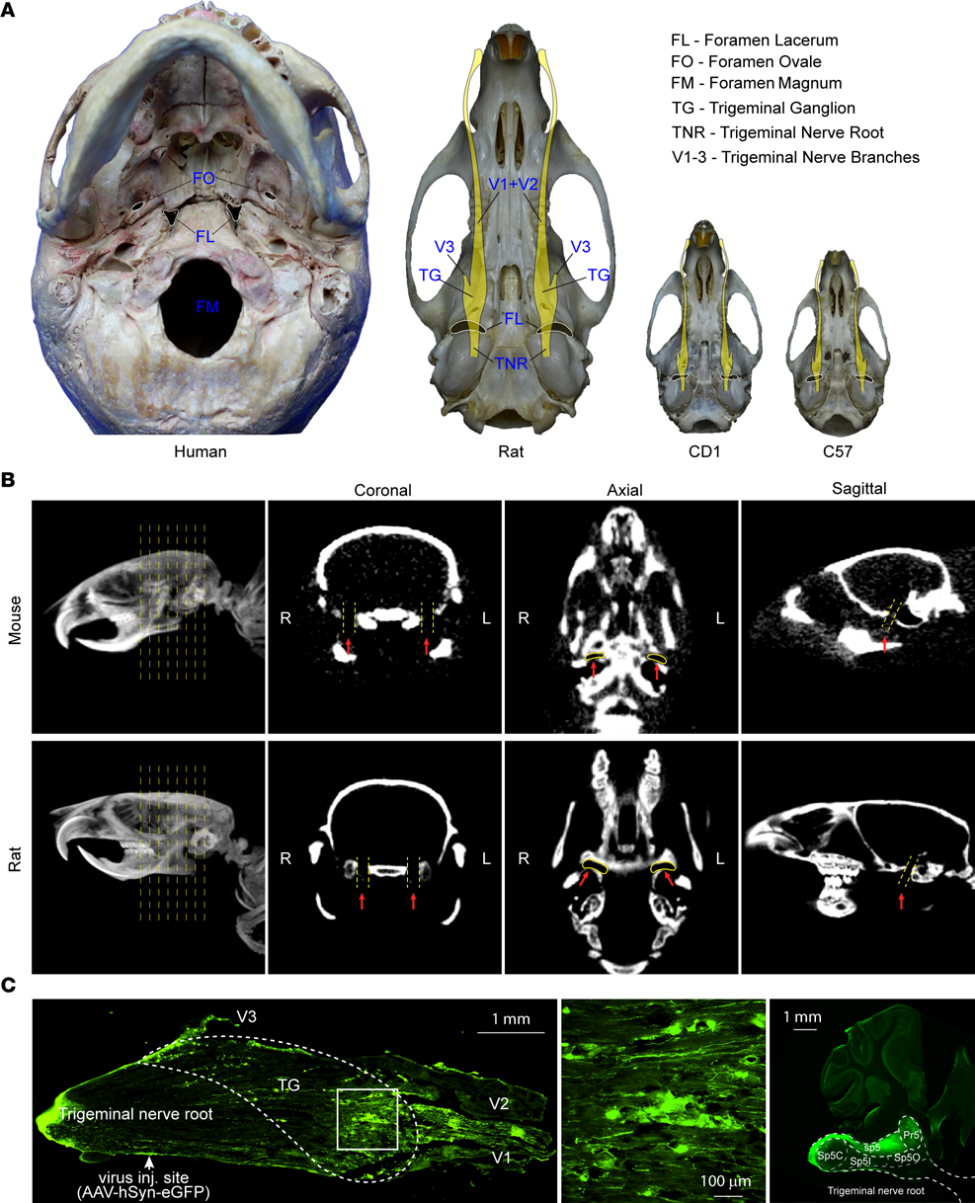
Foramen lacerum anatomy. (**A**) The foramen lacerum in the skull base of human and rodents. Images of human, Sprague-Dawley rat, CD1 mouse, and C57BL/7 mouse skull bases were taken, and schemes of the trigeminal nerve system (yellow color) are overlaid on the skull base of rodents to depict the relationship between the foramen lacerum and trigeminal system. (**B**) CT of the foramen lacerum. Sprague-Dawley rats and C57BL/7 mice were anesthetized and scanned with a CT machine. Three-dimensional reconstruction was performed to obtain coronal, axial, and sagittal views highlighting the foramen lacerum (red arrow). L, left; R, right. (**C**) Virus delivery through the foramen lacerum. AAV1 was directly injected with a pulled glass pipette into the trigeminal nerve root (*n* = 3). The virally expressed GFP is shown for the trigeminal system.

In humans, accessing the trigeminal ganglion through the foramen ovale is routinely used for radiofrequency lesioning to treat TN (12–14). We posited that foramina of the rodent skull base could also be used to access the trigeminal system. For this, we performed anatomical studies in mouse and rat skulls (Figure 1A). We found that in both species the foramen lacerum exhibited an elongated yet slightly slanted shape, located in a relatively posterior position of the skull base. In adult C57BL/6 mice, the foramen lacerum was about 1.97 ± 0.05 mm (mean ± SD) in length and 0.52 ± 0.06 mm in width; in adult Sprague-Dawley rats, it was about 4.04 ± 0.1 mm in length and 1.08 ± 0.05 mm in width (Supplemental Table 1; supplemental material available online with this article; https://doi.org/10.1172/jci.insight.168046DS1).

In humans, skull base foramina can be accessed by facial and neck or submandibular approaches (12, 15). We performed CT scans of mice and rats (Figure 1B) and found that, in both species, the foramen lacerum was a prominent skull base foramen. We then used a cervical midline incision to carefully dissect the muscle and facial layers and were able to find the foramen lacerum in both species under a surgical microscope.

After accessing the foramen lacerum, we sought to examine the neural structure that was immediately accessible through it. We performed microinjection with AAV8-hSyn-eGFP through the foramen lacerum (Supplemental Figure 1, A and B). One month later, robust virus GFP expression was localized in the trigeminal ganglion and trigeminal projection tract in the brain stem (Figure 1C). The injection site was between the trigeminal ganglion and the pons, consistent with the trigeminal nerve root. Additionally, we injected 1% lidocaine through the foramen lacerum. Lidocaine significantly increased facial mechanical withdrawal threshold (Supplemental Figure 1, C and D).

### FLIT induces trigeminal nerve root demyelination.

**Figure 2:**
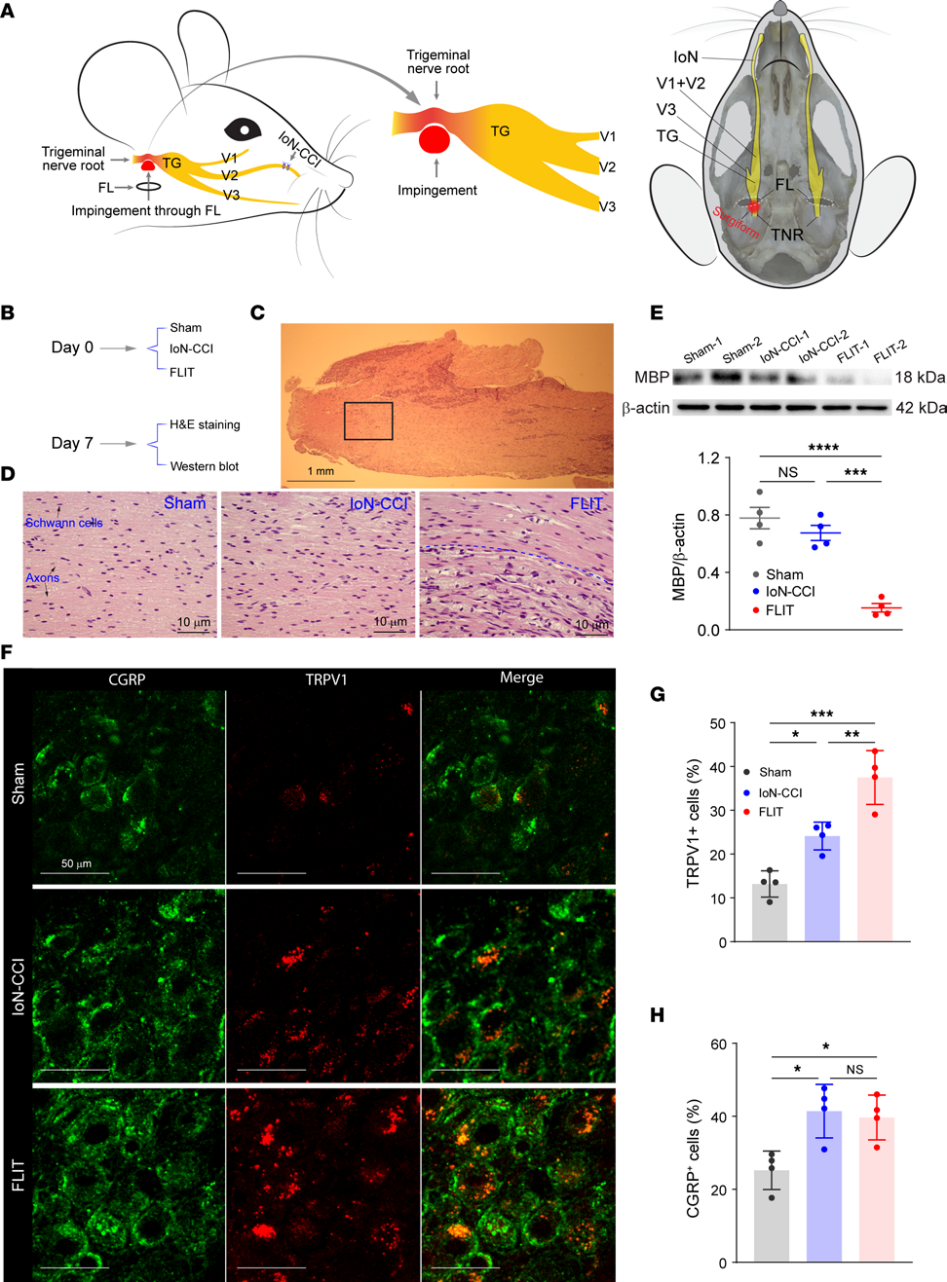
FLIT leads to demyelination of the trigeminal nerve root. (**A**) Diagram of the FLIT and IoN-CCI procedure (left) and diagram of trigeminal nerve root impingement in the skull base (right). (**B**–**D**) H&E staining of the trigeminal nerve root (*n* = 4 per group). (**B**) Diagram of study timeline. (**C**) H&E staining at lower magnification. Boxed area indicates a representative view of the trigeminal nerve root. Scale bar: 1 mm. (**D**) Trigeminal nerve root staining. Representative images of each group are shown. Myelinated fibers and Schwann cells are indicated in the image from the sham group. In the image from the FLIT group, above the dashed line shows normal nerve fibers, and below the dashed line shows demyelinated fibers with inflammatory cell infiltration. Scale bar: 10 μm. (**E**) Western blot using an anti–myelin basic protein antibody. Representative blots are shown from 4 animals per group. Quantification plot shows individual values of each mouse, with mean ± SEM shown in scatter plot. One-way ANOVA test with post hoc Tukey’s test was conducted. ***P < 0.001, ****P < 0.0001. (**F**–**H**) CGRP and TRPV1 staining (*n* = 4 animals per group). (**F**) Representative staining from each group. Scale bar: 10 μm. (**G**) Percentage of TRPV1^+^ cells among all neurons. (**H**) Percentage of CGRP^+^ cells among all neurons. Bar charts represent mean ± SD, with each dot representing 1 animal. One-way ANOVA test with post hoc Tukey’s test was conducted. *P < 0.05, **P < 0.01, ***P < 0.001.

The trigeminal nerve root and ganglion are covered by dense dura structure. Therefore, impingement of trigeminal nerve root from a ventral access through the foramen lacerum might lead to sustained localized pressure. To test this, we placed Surgifoam through the foramen lacerum (Figure 2A and Supplemental Figure 2, A–C). One month after placement, Surgifoam-induced local compression was still present (Supplemental Figure 2D). To characterize the histological and biochemical consequences of trigeminal nerve root impingement, the trigeminal nerve root was stained with H&E and also assessed for its myelination by using an antibody against myelin basic protein. FLIT surgery led to substantial demyelination in the trigeminal nerve root and inflammatory cell infiltration (Figure 2, B–D). The remarkable demyelination associated with the FLIT surgery was evident on both H&E stain and Western blot against myelin basic protein (Figure 2E). Interestingly, when the FLIT surgery was compared with a traditional surgery to induce trigeminal neuropathic pain, infraorbital nerve chronic constriction injury (IoN-CCI) (16, 17), the latter induced only mild but not statistically significant demyelination in the trigeminal nerve root (Figure 2, B–E). At the trigeminal ganglion, nociception-related markers including TRPV1 and CGRP were stained with antibodies. The results showed that the FLIT model was associated with higher percentages of TRPV1- and CGRP-expressing cells in the trigeminal ganglion (Figure 2, F–H).

### FLIT leads to distinct TN-like behavior.

**Figure 3:**
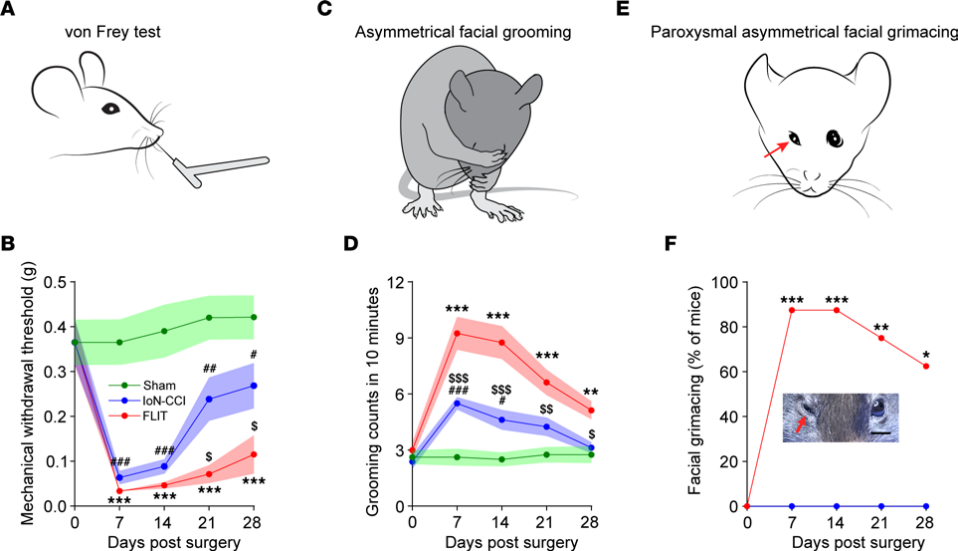
FLIT procedure leads to TN-like behavior. Mice underwent FLIT, IoN-CCI, and sham surgeries (*n* = 8 per group, male), and behavioral characteristics were assessed. Line charts represent mean ± SEM (in color shades), except for **F**. (**A** and **B**) Facial mechanical withdrawal. There was a significant difference among the 3 groups on 2-way ANOVA. Post hoc Bonferroni’s test revealed differences at indicated time points. FLIT versus sham, ***P < 0.001; IoN-CCI versus sham, ^#^*P* < 0.05, ^##^*P* < 0.01, ^###^*P* < 0.001; FLIT versus IoN-CCI, ^$^*P* < 0.05. (**C** and **D**) Asymmetric facial grooming was prominent in the FLIT group. Grooming counts (including asymmetric and symmetric grooming) in 10 minutes were recorded at indicated time points. There was a significant difference among the 3 groups on 2-way ANOVA. Post hoc Bonferroni’s test revealed differences at indicated time points. FLIT versus sham, **P < 0.01, ***P < 0.001; IoN-CCI versus sham, ^#^*P* < 0.05, ^###^*P* < 0.001; FLIT versus IoN-CCI, ^$^*P* < 0.05, ^$$^*P* < 0.01, ^$$$^*P* < 0.001. (**E** and **F**) Paroxysmal asymmetric facial grimacing. Percentages of mice with paroxysmal asymmetric facial grimacing were recorded. Neither the IoN-CCI group nor the sham group displayed this behavior, so only data for the sham group are shown. Fisher’s exact test was used for statistical analysis, *P < 0.05, **P < 0.01, ***P < 0.001.

A set of distinct behavioral phenotypes was observed (Figure 3, A–F, and Supplemental Figure 2E). Because trigeminal nerve root pathologies were implicated in human TN, we wanted to determine whether these phenotypes were reminiscent of clinical presentations and whether these phenotypes were different from those observed in the IoN-CCI model.

Adult C57BL/6 mice underwent either the IoN-CCI or FLIT procedure, and mechanical withdrawal thresholds in the facial area were determined as demonstrated (Figure 3A and Supplemental Video 1). Mice in both models exhibited significant mechanical allodynia (Figure 3B), which lasted longer in the FLIT model. Additionally, mice in the FLIT model displayed significantly more pronounced nociceptive behavior in response to acetone as a cold stimulation (Supplemental Figure 3, A–C). Mice in the FLIT model displayed significant spontaneous facial grooming (Figure 3, C and D, and Supplemental Video 2). Importantly, mice in the FLIT model displayed a unique grooming movement pattern: The strokes were primarily asymmetric and wider ipsilateral to the injury side. More strikingly, mice in the FLIT model displayed paroxysmal asymmetric facial grimacing (Figure 3, E and F, and Supplemental Video 3) in about 65%–80% of animals. These facial grimaces included squinting of the eye on the side of trigeminal injury, intermittent facial twitches, or shaking of whiskers. The paroxysmal asymmetric facial grimacing typically lasted for a few seconds and resolved spontaneously. In humans, TN is also called tic douloureux, with dramatic bouts of lancinating pain and facial tics (3, 4). The paroxysmal intermittent facial grimacing was observed only in the FLIT model, not in the IoN-CCI model.

### Dental pain–like behavior in the FLIT model.

**Figure 4:**
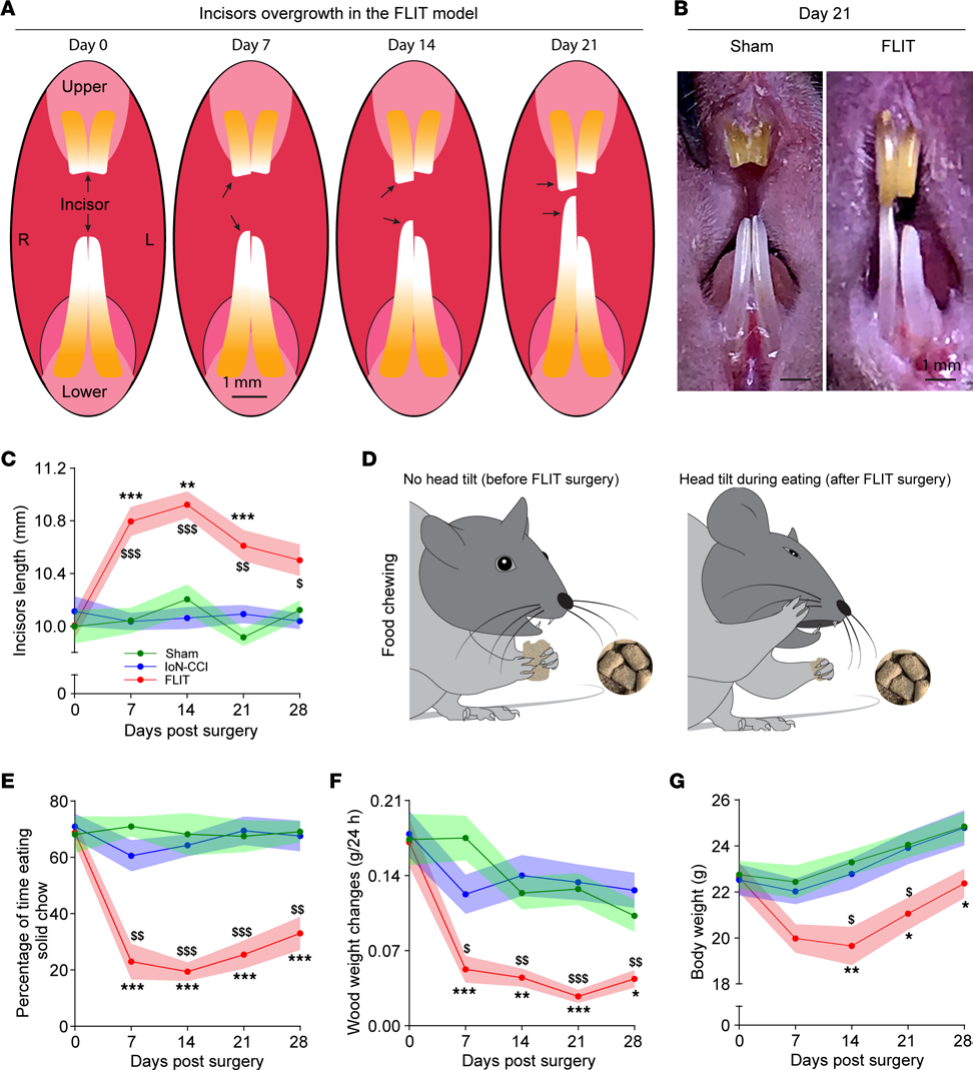
Dental pain–related behavior in the FLIT model. Mice underwent FLIT, IoN-CCI, and sham surgeries (*n* = 8 per group, male). Line charts represent mean ± SEM (in color shades). (**A**) Diagram of incisor overgrowth in the FLIT model. (**B**) Representative image of incisor overgrowth in the FLIT model. (**C**) Incisor length. There was significant difference among the 3 groups on 2-way ANOVA test. Post hoc Bonferroni test revealed differences at indicated time points. FLIT versus sham, **P < 0.01, ***P < 0.001; FLIT versus IoN-CCI, ^$^*P* < 0.05, ^$$^*P* < 0.01, ^$$$^*P* < 0.001. (**D** and **E**) Dental pain–like behaviors during food chewing. (**D**) Diagram of head tilt during eating after the FLIT procedure. Left: No head tilt during food chewing before the FLIT surgery. Right: Head tilt while eating after FLIT surgery. (**E**) Percentage of time eating solid chow was counted in 10 minutes. There was a significant difference among the 3 groups on 2-way ANOVA test. Post hoc Bonferroni’s test revealed differences at indicated time points. FLIT versus sham, ***P < 0.001; FLIT versus IoN-CCI, ^$$^*P* < 0.01, ^$$$^*P* < 0.001. (**F**) Balsa wood weight changes. Mice were singly housed overnight with a balsa wood blocks, and wood weight changes were quantified. There was a significant difference among the 3 groups on 2-way ANOVA test. Post hoc Bonferroni’s test revealed differences at indicated time points. FLIT versus sham, *P < 0.05, **P < 0.01, ***P < 0.001; FLIT versus IoN-CCI, ^$^*P* < 0.05, ^$$^*P* < 0.01, ^$$$^*P* < 0.001. (**G**) Body weight changes after FLIT procedure. There were significant differences between the 3 groups on 2-way ANOVA test. Post hoc Bonferroni’s test revealed differences at indicated time points. FLIT versus sham, *P < 0.05, **P < 0.01; FLIT versus IoN-CCI, ^$^*P* < 0.05.

Mice in the FLIT group grew their teeth at a faster rate when compared with the IoN-CCI and sham groups (Figure 4, A–C). For the IoN-CCI and sham groups, the total length of frontal incisors remained relatively unchanged during the experimental observation periods, whereas the FLIT group displayed poor dentition and excessive dental growth to the point that necessitated manual trimming (Figure 3B). Of note, because of trimming and occasional tooth fracture secondary to poor dentition, reported data probably underestimated incisor length in the FLIT group.

In the FLIT group, eating and chewing were commonly associated with excessive facial grooming and, in many instances, face patting (Supplemental Video 4). More strikingly, mice in the FLIT group often tilted their heads away from the FLIT surgery side (Figure 4D and Supplemental Figure 4, A and B). The head tilt presumably allowed food to be chewed by the contralateral side. This altered eating behavior prompted us to examine whether soft chow would be preferred by these mice. To directly assess food preference between solid and soft chow, mice were provided with both types of food, and eating time for solid versus soft was quantified. At baseline, before surgeries, animals in all groups spent slightly over 50% of eating time on solid chow, suggesting a slight preference for solid chow. This preference was maintained in both the sham and IoN-CCI groups after surgeries. However, mice in the FLIT group spent 70%–80% of eating time on soft chow, consistent with significant preference for soft chow and avoidance of solid chow (Figure 4E). The avoidance of solid chow and observed head tilt during eating were suggestive of dental pain.

To directly assess chewing activities, mice were tested for their chewing of wood blocks. It was found that cork blocks were not associated with consistent chewing, presumably because of the chemical glue used during the manufacturing process. On the other hand, oak, maple, and pine blocks were too hard for C57BL/6 mice to chew. Balsa wood was chosen because it is relatively soft according to the Janka scale of wood hardness (18). When mice in the FLIT, IoN-CCI, and sham groups were individually housed in cages supplied with balsa wood blocks, the wood block weight changes were measured to assess spontaneous chewing. At baseline before surgeries, mice in all groups demonstrated significant chewing as balsa wood blocks were chewed from cube shape to ball shape. This chewing ability was maintained in both the sham and IoN-CCI groups after surgeries. However, in the FLIT group, chewing assessed by balsa wood weight changes was significantly decreased (Figure 4F).

Moreover, mice in the FLIT model group usually lost about 10% of their body weight during the first few days after surgery, followed by slower weight gain when compared with that in mice in the IoN-CCI or sham groups (Figure 4G). Therefore, inadequate weight gain, head tilt when eating, avoidance of solid chow, and lack of wood chewing were all consistent with dental pain–like behavior, a common clinical presentation of TN in humans.

We also determined the duration of pain-like behavior in the FLIT model and found that many of the behaviors resolved in about 5–6 weeks (Supplemental Figure 4, C–F).

### Optimizing Surgifoam-induced compression in the FLIT model.

**Figure 5:**
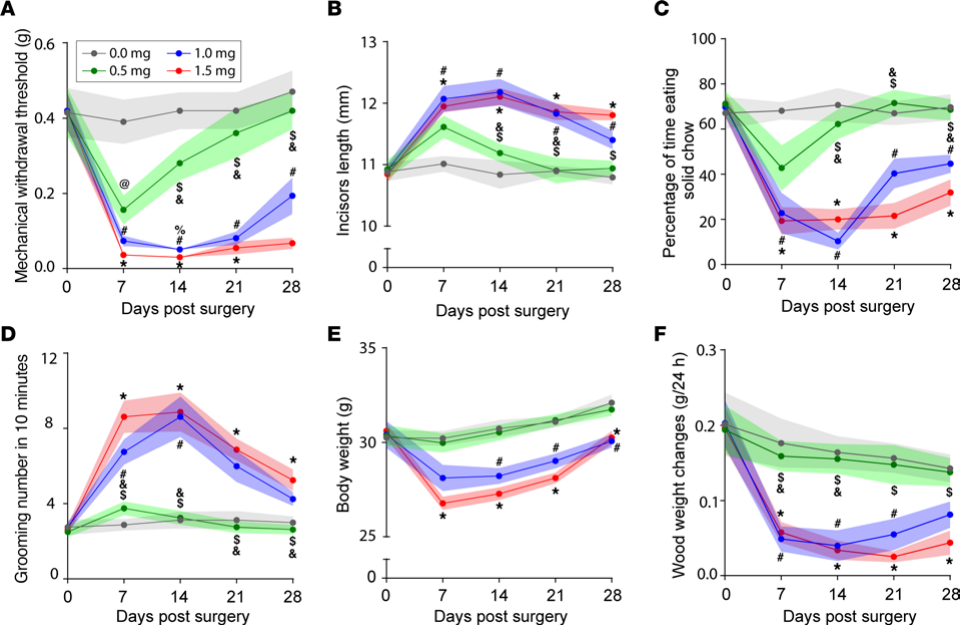
Doses of Surgifoam and pain-like behavior. Mice (male) underwent FLIT procedure with different doses of Surgifoam (0.0 mg sham, 0.5 mg, 1.0 mg, and 1.5 mg), *n* = 8 per group. Behavioral testing was performed at indicated time points. Line charts represent mean ± SEM (in color shades). (**A**) Mechanical withdrawal threshold. (**B**) Incisor length. (**C**) Percentage of time chewing solid chow. (**D**) Grooming counts in 10 minutes. (**E**) Body weight changes. (**F**) Wood weight changes in 24 hours. There was a significant difference among the 4 groups on 2-way ANOVA test. Post hoc Bonferroni’s test revealed differences at indicated time points; all symbols represented *P* < 0.05. Specific comparisons: *, 0 mg versus 1.5 mg; $, 0.5 mg versus 1.5 mg; @, 0 mg versus 0.5 mg; %, 1.0 mg versus 1.5 mg; #, 0 mg versus 1.0 mg; and &, 0.5 mg versus 1.0 mg.

To refine and optimize the FLIT surgery, different doses of Surgifoam were compared in adult C57BL/6 mice. Specifically, 0.5 mg, 1.0 mg, and 1.5 mg Surgifoam doses were used for FLIT surgery, and all these behavioral endpoints were compared (Figure 5, A–F). At 0.5 mg, Surgifoam induced relative mild mechanical allodynia, which started to resolve within 2 weeks after surgery (Figure 5A). Similarly, this dosage induced only mild tooth overgrowth (Figure 5B) and a tendency to mild avoidance of solid food (Figure 5C), without significant changes in grooming (Figure 5D), body weight gain (Figure 5E), or wood chewing (Figure 5F). On the other hand, both 1 mg and 1.5 mg induced mechanical allodynia (Figure 5A), incisor overgrowth (Figure 5B), avoidance of solid chow (Figure 5C), excessive grooming (Figure 5D), inadequate weight gain (Figure 5E), and lack of wood chewing (Figure 5F). There was a modest tendency to more severe pain-like behavior with 1.5 mg than 1.0 mg. However, the difference between these 2 groups did not reach statistical significance for the majority of time points. Based on these results, 1.0–1.5 of Surgifoam produced desirable behavioral endpoints for adult C57BL/6 mice.

### FLIT model in CD1 mice and in Sprague-Dawley rats.

**Figure 6:**
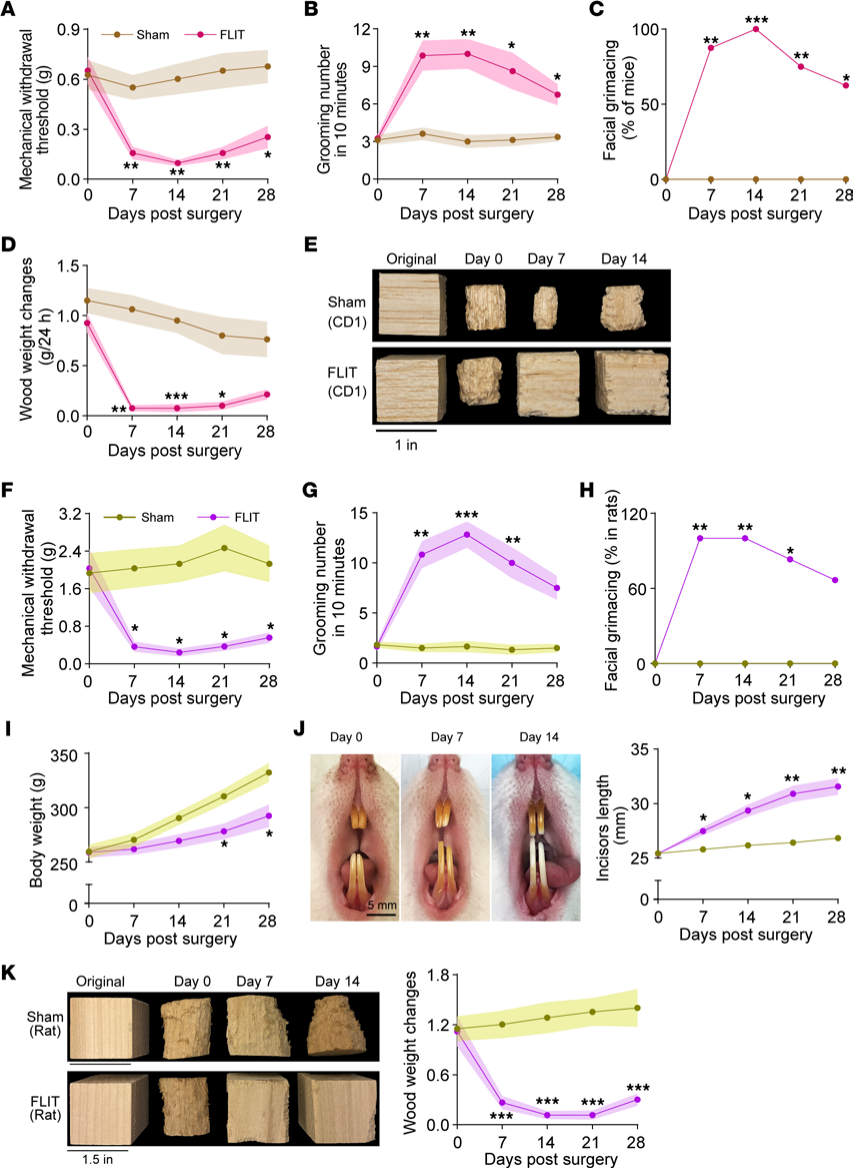
FLIT model in CD1 mice and Sprague-Dawley rats. (**A**–**E**) FLIT model in CD1 mice. CD1 mice underwent FLIT or sham procedure (*n* = 8 per group, male) and were assessed for behavior: (**A**) facial mechanical withdrawal threshold, (**B**) grooming counts in 10 minutes, (**C**) percentage of mice with facial grimacing, (**D**) balsa wood weight changes, and (**E**) representative images of balsa wood blocks in sham and FLIT groups. (**F**–**K**) FLIT model in male Sprague-Dawley rats. Sprague-Dawley rats underwent FLIT or sham procedure (*n* = 6 per group) and were assessed for behavior: (**F**) facial mechanical withdrawal threshold, (**G**) grooming counts in 10 minutes, (**H**) percentage of mice with facial grimacing, (**I**) body weight, (**J**) representative images of incisors in rats that underwent FLIT and incisor length, and (**K**) representative images of pine wood blocks in sham and FLIT groups and wood block weight changes. Line charts represent mean ± SEM (in color shades) except for **C** and **H**. Two-way ANOVA test was used for panels **A**, **B**, **D**, **F**, **G**, **I**, **J**, and **K**, and there was a statistically significant difference between groups. Post hoc Bonferroni’s test was used to determine the time points at which the difference existed. FLIT versus sham, *P < 0.05, **P < 0.01, ***P < 0.001. Fisher’s exact test was used for panels **C** and **H**, *P < 0.05, **P < 0.01, ***P < 0.001.

The foramen lacerum anatomy appeared to be conserved in different strains of mice and between mice and rats. The FLIT model was tested in CD1 mice and in Sprague-Dawley rats. In CD1 mice, mechanical allodynia (Figure 6A), increased grooming (Figure 6B), paroxysmal asymmetric grimacing (Figure 6C), and lack of wood chewing (Figure 6, D and E) were observed. In Sprague-Dawley rats, mechanical allodynia (Figure 6F), increased grooming (Figure 6G), percentage of rats with facial grimacing (Figure 6H), inadequate body weight gain (Figure 6I), incisor overgrowth (Figure 6J), and lack of wood chewing (Figure 6K) were observed. These results extended the FLIT model into different strains of mice and different species. However, there were notable behavioral features unique to different animal strains and species. For example, facial grooming tended to be more aggressive in CD1 than C57BL/6 mice, which led to facial skin ulcer in about 30% of mice tested. Wood chewing activities were also more readily observed across different types of wood blocks in Sprague-Dawley rats, including pine blocks and balsa wood, presumably related to different jaw strengths in different species.

### Robust activation of the primary somatosensory cortex in the FLIT model.

**Figure 7:**
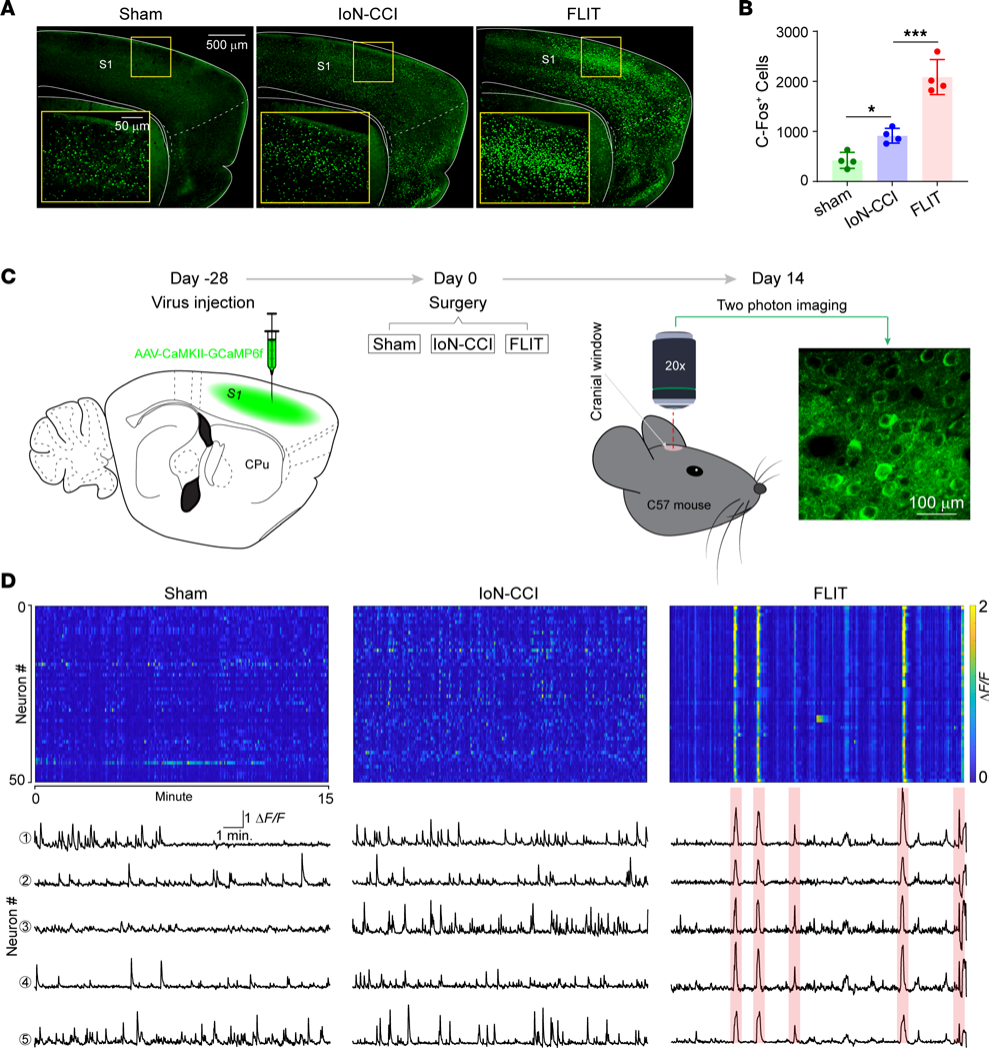
Unique cortical neural activation pattern in the FLIT model. (**A** and **B**) *c-Fos*-positive cells in the FLIT model. Mice (*n* = 4 per group) underwent FLIT, IoN-CCI, and sham surgeries. Two hours after surgery, brains were obtained for *c-Fos* staining. (**A**) Representative *c-Fos* staining in different groups. (**B**) Number of *c-Fos*–positive cells in the S1 cortex. One-way ANOVA indicated statistically significant differences present between groups. Post hoc Tukey’s test was performed. *P < 0.05, ***P < 0.001. (**C** and **D**) Two-photon calcium imaging in awake and resting state. Mice (*n* = 4 per group) underwent S1 cortex microinjection of AAV8-CaMKII-GCaMP6f and were allowed 4 weeks for virus expression. Two-photon calcium imaging was performed without anesthesia at a resting state. (**C**) Experimental design. Left sketch depicts virus injection in S1 of mouse followed by surgeries at day 0. The panel on the far right is a representative image taken during 2-photon imaging session to demonstrate GCaMP6f expression. (**D**) Top panels are heatmaps of calcium dynamics. For all heatmaps, 50 neurons are shown over 15 minutes of imaging time. Color represents Δ*F/F*: blue color, lower values of Δ*F/F*; yellow color, higher values of Δ*F/F*. Lower panels show representative calcium tracing from the imaging field. Five representative single neurons are shown. Red color indicates synchronized firing.

The cerebral cortex is at the top of the brain’s hierarchy for information integration, including sensory information (19). The primary somatosensory cortex (S1) is important for pain processing (20–22). To study the S1 activation in different pain models, mice in the FLIT, IoN-CCI, and sham groups were sacrificed 2 hours after surgery. Brain slices were stained for *c-Fos* expression as a marker for neural activation. The number of *c-Fos*–positive cells in the S1 cortex was significantly higher in the FLIT model than the IoN-CCI model (Figure 7, A and B). To determine the neural activity patterns, the S1 cortex was microinjected with AAV8-CaMKII-GCaMP6f followed by 4 weeks of resting to allow optimal viral expression (Figure 7C). GCaMP6f reliably detects single action potentials in neuronal somata and synaptic calcium transients in individual dendritic spines, which makes it a powerful tool in dissecting neuronal activities as well as dynamics of neural circuits over multiple spatial and temporal scales (23–28). Using intravital 2-photon calcium imaging in nonanesthetized mice at resting state, we recorded contralateral S1 neural population dynamics at single-cell resolution before (Supplemental Figure 5A) and after surgery. Mice in the FLIT group displayed synchronized neural activities as previously reported (11), which were absent in the IoN-CCI and sham groups (Figure 7D). The S1 neuronal synchronization was confirmed with *VGlut2-Cre* mice and AAV8-EF1a-DIO-GCaMP6f (Supplemental Figure 5, B and C). Besides robust synchronization at resting state, the FLIT model also displayed heightened S1 activity against mechanical stimuli (Supplemental Figure 5, D–F). Therefore, the upregulation of *c-Fos* was accompanied by a unique S1 neuronal activity pattern in the FLIT model.

To examine S1 neural plasticity, *Thy1-YFP* mice underwent FLIT, IoN-CCI, and sham surgeries. Postsynaptic dendritic spines on apical dendrites of layer 5 pyramidal neurons of the S1 were observed. We observed dendritic spine turnover, and spine elimination was significantly increased at day 7 after FLIT or IoN-CCI surgery (Supplemental Figure 6, A–C). Additionally, dendritic spine elimination appeared to be more pronounced in the FLIT model than the IoN-CCI model (Supplemental Figure 6B), along with comparable spine formation between the 2 groups at day 7 after surgeries (Supplemental Figure 6C).

## Discussion

Encouraged by previous work to recapitulate human TN in animal models (9, 10, 29), we report an anatomy-based approach to take advantage of a natural foramen in the skull base to reliably access the trigeminal nerve root. The procedure, named FLIT, induced behavioral phenotypes that were consistent with key clinical features of TN. For example, facial tics in humans were recapitulated with paroxysmal asymmetric facial grimacing. Dental pain is a common clinical presentation of TN, and therefore many patients are misdiagnosed with dental diseases (30, 31). The FLIT model was associated with several unique features that, when considered collectively, were probably related to dental pain. For example, inadequate weight gain could be due to other causes. However, when considered in conjunction with lack of wood chewing and avoidance of solid food, it was probably related to dental pain. Consistent with dental pain–like behavior, head tilting away from the trigeminal injury side was present. The tilt presumably facilitated chewing food by using teeth on the uninjured side (“no-pain” side). Moreover, tooth overgrowth was also indicative of lack of chewing or altered chewing, consistent with dental pain–like behavior. In humans, the oral trigger zone is commonly present in TN (3, 4). Because of the dental pain–like behavior, we suggest placing softened food on the cage floor to increase nutritional support in the FLIT model. Of note, although behavior data reported in this article were based on male mice and rats, similar results were observed in female mice and rats (data not shown).

Clinically, the trigeminal ganglion is accessed for radiofrequency lesioning or balloon pump compression through the foramen ovale (3, 32, 33). It is usually accessed via the Hartel approach from the upper molar level in the face (15, 34). A submandibular approach to access the foramen ovale has been described (12). In rodents, the foramen ovale has not been consistently described (35, 36). In this report, we showed that the foramen lacerum was easily identifiable in rodents. Using CT scans, we found that its relatively caudal location could facilitate access through a small neck incision. The trigeminal nerve root was directly dorsal to the medial aspect of the foramen lacerum. We reported direct gene delivery to the trigeminal system through the foramen lacerum, establishing a route for gene therapy.

Mechanical allodynia was present in the orofacial area in both the IoN-CCI and FLIT models. Although reflex-based evoked pain behaviors have been widely used for pain assessment in research, pain in humans often presents as spontaneous pain in “nonevoked” conditions, in the absence of externally imposed stimuli (37). Recapitulating spontaneous pain-like behavior, without experimentally imposed stimuli, has gained significant research interest (37, 38). For example, facial grimacing scale has been widely accepted as a facial expression coding of pain (39, 40). Notably, the facial grimacing scale, including in the IoN-CCI model, involved bilateral symmetric facial grimaces (41). In the present study, the facial grimaces were asymmetric, involving squinting of the eye and facial tics on the ipsilateral side of the injury. The asymmetric facial grimacing was unlikely to be caused by permanent facial nerve damage, because it was typically paroxysmal and resolved spontaneously, accompanied by facial tics. The trigeminal nerve is a mixed sensory and motor nerve, with the latter innervating masseter muscles. Using botulinum toxin injection to induce unilateral masseter muscle atrophy, we did not observe significant dental pain–like behavior or facial mechanical allodynia (11). Therefore, the behavior phenotypes of the FLIT model were unlikely to be caused by inadvertent motor damage to the trigeminal nerve.

The FLIT model can be applied to both mice and rats, accommodating a range of experimental designs. Despite their outbred background, CD1 mice have been widely used in neuroscience studies because they are inexpensive, robust, and readily available (42–44). Interestingly, despite largely overlapping behavioral phenotypes across strains and species, there were some noticeable differences. One difference was that CD1 mice displayed self-mutilation due to excessive facial scratching. Severe self-mutilation or autotomy has been reported from rodents to nonhuman primates after nerve injury (45), and strain differences have been described in mice. For example, after sciatic and saphenous nerve injury, BALB/c and C3H strains were more likely to display self-mutilation than C57BL/6 and C57BL/10 strains (46), presumably related to genetic differences.

Many of the behaviors reported in the FLIT model, such as facial grimacing, wood chewing, and food preference, could be assessed in animals’ home cages. Environmental factors, including those associated with behavioral testing, are known to confound pain studies (47, 48). The naturalistic setting of the home cage could, in theory, increase the reliability of these assays. More importantly, many of these assays were easily quantifiable, providing objective and quantitative measurements related to pain.

One key feature of the FLIT model was its reversibility. After a decompressive surgery to remove the Surgifoam, pain-like behavior in the FLIT model could largely resolve (11). In humans, microvascular decompression is a curative treatment in many cases of TN (3, 49). The resolution of TN-like symptoms after decompression in the FLIT model not only supports the validity of the model but also suggests an important contribution of peripheral inputs in TN. In line with peripheral inputs, dorsal root ganglion inputs were found to be instrumental in the synchronization of S1 (50). Interestingly, the FLIT model displayed both heightened TRPV1 and CGRP expression in the trigeminal ganglion and increased S1 dendritic spine turnover, suggesting a link between peripheral inputs and central neural plasticity.

It is likely that interneuron hypoactivity underlaid the observed cortical synchrony (11). A substantial amount of work has established dense interconnectivity between excitatory and inhibitory neurons in S1 and sensory cortices in general (51–55). Furthermore, a link between inhibitory neuron hypoactivity and excitatory neuron hyperactivity has been demonstrated (56, 57). However, the specific mechanisms through which interneuron hypoactivity lead to excitatory neuron hypersynchronization are poorly understood. This synchronization mimics focal seizure activity. Carbamazepine, an antiseizure drug and a first-line medication for TN, attenuated TN-like behavior and cortical synchronization in the FLIT model (11). Besides central mechanisms, it is likely that peripheral inputs are also implicated (as shown in refs. 11, 50).

Trigeminal neuropathic pain has been successfully modeled in the IoN-CCI model (16, 17), whereas modeling TN proved challenging in rodents (58). By impinging the trigeminal nerve root through the foramen lacerum, the FLIT model captures characteristic clinical features of TN. Using the FLIT model, we discovered robust cortical S1 activation and neural dynamics that were different from those of the IoN-CCI model, unraveling unique neural dynamics of TN. Therefore, the FLIT model provides an additional tool for disease-specific mechanistic studies and testing of analgesics.

## Methods

### Animals.

We purchased 4- to 5-month-old male and female C57BL/6, *Thy1-YFP* line (strain 003782) and *VGlut2-Cre* mice (strain 028863) from The Jackson Laboratory. Mice were housed in temperature-controlled vivarium on a 12 hours light/dark cycle (lights on at 07:00 am, lights off at 07:00 pm) with food and water available ad libitum. CD1 mice (16–26 weeks old, male) and Sprague-Dawley rats (10–14 weeks old, male) were also purchased from the Jackson Laboratory and Charles River Laboratories, respectively.

### CT scan.

Rodents were anesthetized with isoflurane inhalation and placed in prone position with neck extended in Triumph rodent X-O scan (GE Healthcare). Sagittal, coronal, and oblique views were shown in PMOD software (PMOD Technologies, LLC) reconstruction of CT images.

### FLIT procedure.

Mice were anesthetized with isoflurane inhalation (3% for induction and 1.5%–2% for maintenance) in oxygen (flow rate, 1–2 L/min). The surgery was performed under an Omano surgical microscope (OM2300S-V7) at ×7–×45 magnification. After surgical field preparation, a 1 to 1.5 cm midline neck incision was made starting at the rostral end of the sternum with a sterile spring scissor. The superficial tissues such as salivary glands were bluntly dissected and lateralized with a mini retractor. The neck muscles, including the sternocleidomastoid muscle, digastric muscle, and strap muscles, were gently dissected to locate the auditory bulla and auditory capsule on the right side of the mouse head, which are the landmarks for locating the foramen lacerum. Electric cauterization was applied to control minor bleeding from capillaries. Care was taken while dissecting the vessels covering both the anterior bulla and the foramen lacerum. A prepared piece of Surgifoam (Ethicon Inc.) at approximately 1–1.5 mg (mice) or approximately 2.5 mg (rats) was gently delivered into the foramen lacerum with curved forceps. The Surgifoam was positioned between the trigeminal nerve root and the cochlear bulla. After removing the retractor and replacing the tissues, we closed the skin with 4-0 nylon monofilament (Ethicon Inc.) sutures. Animals in the sham group underwent the same surgical procedure, including neck shaving, skin incision, muscle dissection, and foramen lacerum exposure without physical trigeminal nerve root compression. The surgery time ranged from 8 to 12 min per animal. A representative video demonstrating FLIT surgery was included (Supplemental Video 5).

### Infraorbital nerve chronic constriction injury.

The IoN-CCI procedure was performed as previously described (17) with minor modifications. Briefly, mice were anesthetized with isoflurane inhalation (3% for induction and 1.5%–2% for maintenance) in oxygen (flow rate, 1–2 L/min). The skin between the eye and facial whiskers was prepared. A 0.5 cm incision parallel to the midline was made from the caudal end of the third to fourth row of facial whiskers to the ipsilateral inner canthus. The immediately exposed superficial tissues were bluntly dissected and lateralized with a mini-retractor. When the trunk of the infraorbital nerve was exposed outside the inferior orbital fissure, 2 chromic gut ligatures (6-0) were loosely placed around the distal segment of infraorbital nerve 2 mm apart.

### Mechanical withdrawal threshold.

Animals were individually placed in a custom-made testing enclosure allowing for free head movement. After 30 minutes of acclimation, a graded series of von Frey filaments were inserted through the mesh walls from the lateral side and applied to the skin of the vibrissa pad within the trigeminal nerve V2 branch-innervated territory for 1 second at 10-second intervals. A brisk withdrawal of the head upon stimulation was considered a positive response. Animals were tested 5 times, with at least 3 positive responses indicating a positive result. The minimum force necessary to elicit a response was defined as the mechanical withdrawal threshold.

### Observation of face grooming and grimacing.

For the facial grooming and grimacing test, each animal was habituated 30 minutes daily for up to 3 consecutive days in a Plexiglas box equipped with a mirror to record unobstructed views of the orofacial area. The mouse behaviors were recorded for 10 minutes without any extra audio or physical disturbance. Grooming was defined as face wash strokes directed primarily to the trigeminal nerve impingement side. Facial grimacing for this study was defined as asymmetric eyelid contraction such that the ipsilateral eye (same side as trigeminal nerve compression) opening was smaller than the contralateral side, as determined by masked observers. The recorded behaviors were analyzed by an experimenter who was masked to the procedure condition and group assignment of the mice.

### Food preference.

Animals were deprived of food 12 hours before the test, with water accessible ad libitum. To prepare the soft chow, regular solid chow was soaked in water (1:2 pellet/water ratio) for 20 minutes. Regular solid chow and freshly prepared soft chow were placed in plates. The test mouse was videotaped with a camera placed 40 cm above the cage for 10 minutes. Time spent eating solid and soft chow in each video was quantified by experimenters who were masked to group assignment.

### Wood chewing assay.

Balsa wood blocks were cut into 1-inch cubes. Mice were housed in individual cages with food and water supplied ad libitum, and a balsa wood block was placed in the cage for 24 hours. Blocks were weighed before and after placement. For rats, pine wood blocks were cut into 1.5-inch cubes. Two rats were cohoused in a cage, with food and water supplied ad libitum, and a wood block was placed in the cage for 7 days. Blocks were weighed before and after placement.

### Acetone test.

Mice were placed in acrylic glass enclosures on an elevated metal mesh and allowed to acclimate the testing room 30 minutes daily for 3 days before testing. After the mice calmed down, 20 μL acetone (Fisher Scientific) was applied with a micropipette to the lower jaw or whisker pad ipsilateral to trigeminal nerve injury side. The animals exhibited multiple responses to the acetone application. We assigned an acetone score, with 0 indicating no response, 0.5 indicating brushing, 1 indicating grimacing or flinching, 2 indicating brushing and grimacing, 3 indicating flinching and brushing or flinching and grimacing, and 4 indicating flinching, brushing, and grimacing. Behavior was observed during the first 60 seconds after acetone application, and measurements were repeated 2 times with a 10-minute interval to obtain a mean value. We tested all animals in the V2 and V3 branch of trigeminal nerve innervated areas, respectively, and spared the V1 branch due to eye irritation and animal welfare.

### Craniotomy and virus injection.

Cranial windows were implanted on the contralateral side to FLIT, IoN-CCI, and sham procedure side in mice. Mice were anesthetized with isoflurane (3% for induction and 1.5% for maintenance in oxygen). The eyes were moistened with eye lubricant. To minimize postoperative pain, ketorolac tromethamine (Athenex) was administrated (5 mg/kg) intraperitoneally every 12 hours for 3 consecutive days. The fur on the top of head was shaved between the outer canthus and concha, and the mouse was positioned in a stereotactic frame with a head holder. The skin was prepared with povidone-iodine solution (Aplicare, Inc.) followed by 70% alcohol swab (BD). After lidocaine (0.2 mL, 1%) infiltration, a skin flap overlying the dorsal skull was removed with microscissors. Connective tissues and periosteum of the parietal skull were thoroughly cleaned. A 3- × 3-mm piece of bone was removed to reveal the left anterolateral cortex, including the primary somatosensory cortex, as determined by stereotactic coordinates following Chen et al. (59), and the dura was kept moist with sterile saline.

For GCaMP6f expression in pyramidal neurons, adeno-associated virus AAV8 carrying CaMKII-GCaMP6f (pENN.AAV.CamKII.GCaMP6f.WPRE.SV40, Addgene 100834, 1 × 10^12^ genome copies/mL) was injected with a Nanoject III (Drummond Scientific Company, model 3-000-207) at a depth of 200 μm beneath the pia surface, and the virus was slowly injected at 4 or 5 sites approximately 1–4 mm lateral to the midline of the skull and approximately1 to –2 mm from the bregma in WT mice. For GCaMP6f expression in Vglut2-Cre mice, AAV8-EF1a-DIO-GCaMP6f (a gift from Guoping Feng’s lab, Massachusetts Institute of Technology) was injected into the contralateral side of the S1 cortex. For *Thy1-YFP* mice, a glass window was implanted instantly after craniotomy.

### Two-photon imaging.

Before the imaging session, mice were taken to the 2-photon microscope room in a head fixation device for 30 minutes daily, for more than 3 sessions for habituation. In vivo imaging was performed with a 2-photon system (Ultima; Bruker) equipped with a Mai Tai laser (Spectra-Physics, KMC 100). The laser was tuned to 910 nm, and the average laser power through the transcranial window was approximately 20–30 mW for imaging acquisition with a 20×, 1.0 NA water immersion objective (Olympus). All images were acquired at frame rate of 6–12 Hz with Prairie View software in awake status without anesthesia. Of note, for any given animal, the field of view was kept constant to obtain images on the same group of neurons longitudinally. For dendritic spine imaging, baseline was acquired before surgery, and repeated imaging was acquired at day 7 after surgery. All dendrite views were imaged with *Z*-stacks. Quantifications of dendritic spines were adopted from Yang et al. (60).

### Calcium imaging data analysis.

Imaging data were corrected for motion between frames in the NoRMCorre software package (61). Neuron selection was carried out subsequently with custom-written software in MATLAB (Mathworks). Calcium fluorescence signals of each individual neuron were extracted from the corrected video files. We corrected the signal for each neuron for background fluorescence changes by subtracting the fluorescence changes from the immediate surrounding. Each neuron’s activity time course was then quantified with the formula Δ*F* = (*F* – *F0*)/*F0,* where *F* is the fluorescence signal at a given frame and *F*0 was calculated from a sliding window of ±30 seconds around the frame. Finally, baseline correction was carried out by fitting a linear function (MATLAB function robustfit) to the lowpass filtered (cutoff, 0.3 Hz) signal. A deconvolution algorithm (fast online deconvolution of calcium imaging data) was applied to detect transients (62). The start and end of transients were detected when the model was above 0.1. For responsive neurons in the mechanical stimulation test, an increase of neuronal activity of at least 25% from a neuron’s own baseline within 10 seconds of stimulation was used as a cutoff.

### H&E staining.

Frozen trigeminal ganglion tissues were sliced (5 μm) and mounted to slides to visualize ganglion and trigeminal nerve structures. Briefly, slides were incubated in Harris’s hematoxylin for 5 minutes, rinsed in H_2_O for 5 minutes, and differentiated in acid alcohol (1% HCl in 70% ethanol) for 10 seconds. Next, sections were rinsed with distilled H_2_O (dH_2_O) for 5 minutes and incubated in ammonia water (0.25% ammonia hydroxide in dH_2_O) for 3e5 minutes and rinsed with dH_2_O twice (5 minutes). Sections were dipped in alcoholic eosin for 10 seconds, rinsed with dH_2_O twice (5 minutes), dehydrated in ethanol, and then mounted onto coverslips.

### Immunofluorescence staining.

Mice underwent surgeries (sham, IoN-CCI, and FLIT) and were briefly maintained under isoflurane anesthesia, and procedures were performed after lidocaine 1% infiltration of the incision sites. Two hours after surgery, mice were sacrificed and perfused with ice-cold PBS followed by 4% paraformaldehyde in 0.1 M phosphate buffer (4% PFA). Brain samples were fixed in 4% PFA at 4°C for 24 hours. Coronal brain sections were sliced at 60 μm thickness with a Leica vibratome (VT1000 S). Tangential slices covering the primary somatosensory cortex was used for *c-Fos* staining. Slices were washed in PBS 5 minutes 3 times, followed by blocking with 6% goat serum and 2% BSA in PBS with 0.3% Triton X-100 (blocking solution) at room temperature for 1 hour. Floating slices were stained with primary antibody (rabbit anti–*c-Fos*; Cell Signaling Technology, catalog 2250, 1:500 dilution) in blocking solution at 4°C overnight. After washing with PBS 5 minutes 3 times, slices were incubated with secondary antibody (goat anti-rabbit Alexa488; Jackson Immunoresearch, catalog 111-545-144). Images were acquired with a Nikon A1 confocal microscope equipped with ×20 and 4× objectives. The acquired images were analyzed in ImageJ (NIH open-source software). Positive cells were counted by an experimenter who was blinded to group assignment. For each group, 4 animals were used. *c-Fos*–positive cells were counted within the slices covering the primary somatosensory cortex individually.

### CGRP, TRPV1, and NeuN staining.

Brain slices were frozen sectioned at 5 μm thickness and incubated with primary antibodies at 4°C overnight, followed by secondary antibody incubation at room temperature for 1 hour. Images were acquired with a Nikon AXR confocal microscope. Guinea pig anti-TRPV1 (Invitrogen, catalog PA129770, 1:1,000 dilution), rabbit anti-CGRP (Immunostar, catalog 24112, 1:500), donkey anti-mouse NeuN (Cell Signaling Technology, catalog 94403, 1:500 dilution), goat anti-rabbit Cy3 (Jackson Immunoresearch, catalog 111-165-003, 1:2,000 dilution), goat anti-mouse DyLight 405 (Jackson Immunoresearch, catalog 115-475-003, 1:250 dilution), and goat anti-guinea pig Cy3 (Jackson Immunoresearch, catalog 106-165-003, 1:2,000 dilution) were used.

### Western blot.

Mice subject to sham, IoN-CCI, and FLIT surgery were sacrificed and trigeminal ganglions were collected at day 14. Proteins were extracted from tissues, and the total protein concentration between samples was equalized. Then, 30 μg protein of each sample was loaded and separated by SDS-PAGE and transferred to a polyvinylidene fluoride membrane (Bio-Rad Laboratories). The primary antibodies of anti–myelin basic protein (Cell Signaling Technology, catalog 83683, 1:1,000) or β-actin (Abcam, catalog 8226, 1:5,000) were incubated in blocking buffer at 4°C overnight. Secondary antibodies (Jackson Immunoresearch, catalog 115-035-003, 1:5,000) were incubated for 1 hour at room temperature. Images were acquired with a ChemiDoc MP imaging system (Bio-Rad Laboratories).

### Statistics.

Data were expressed as mean ± SEM for Figures 3–7, Supplemental Figure 1, and Supplemental Figure 4, C–F and as mean ± SD for Figure 2, Supplemental Figure 3, Supplemental Figure 4, A and B, and Supplemental Figure 5E. The difference in pain behaviors was analyzed via a repeated-measures 2-way ANOVA. Post hoc comparisons with Bonferroni’s corrections were used for comparison across groups at indicated time points. Percentages of mice with facial grimacing were compared across groups via 1-sided Fisher’s exact test. One-way ANOVA followed by Tukey’s post hoc comparison was used for comparison of the 3 groups. Two-tailed unpaired *t* tests were used for 2-group comparisons. A *P* < 0.05 was considered statistically significant. Statistical analysis was carried out in GraphPad Prism software (version 8.0).

### Study approval.

All animal use and procedures followed protocols approved by the Massachusetts General Hospital Institutional Animal Care and Use Committee. Experiments complied with the guidelines established by NIH and the International Association for the Study of Pain.

### Data availability.

All original behavioral and imaging data are available upon request. Two-photon imaging analysis code can be accessed at https://github.com/harnett/Shiqian-analysis (commit 12fa87b).

## Author contributions

WD and SS provided conceptualization. WD, LY, QC, KH, CW, JM, and SS provided methodology. WD, LY, KH, YL, and EB provided investigation. WD, LY, KH, and YL provided formal analysis and data curation. WD and SS wrote the original draft of the manuscript. SS acquired funding.

## Supplementary Material

Supplemental data

Supplemental video 1

Supplemental video 2

Supplemental video 3

Supplemental video 4

Supplemental video 5

Supporting data values

## Version 1 05/09/2023

In-Press Preview

## Version 2 06/08/2023

Electronic publication
