# Supplementary material for: Foramen lacerum impingement of trigeminal nerve root as a rodent model for trigeminal neuralgia
Source: JCI Insight. 2023 Jun 8;8(11):e168046. doi: 10.1172/jci.insight.168046 (PMC10393239; doi:10.1172/jci.insight.168046)
Supplement: Supplemental data [file jciinsight-8-168046-s221.pdf]

Supplementary Table 1

C57BL/6 mice (n=8, mm)									Mean	SD
Length	1.95	2.02	2.05	1.88	1.95	2.01	1.96	1.95	1.94	0.05
Width	0.46	0.52	0.55	0.65	0.48	0.53	0.45	0.52	0.52	0.06
SD rats (n=8, mm)										
Length	3.92	4.11	4.00	3.95	4.01	3.99	4.12	4.22	4.04	0.10
Width	1.02	1.11	1.05	1.06	1.08	1.12	1.06	1.18	1.09	0.05

Supplementary Table 1: Measurements of the foramen lacerum. The Foramen lacerum was measured in both mice and rats (n=8 each species). SD: standard deviation

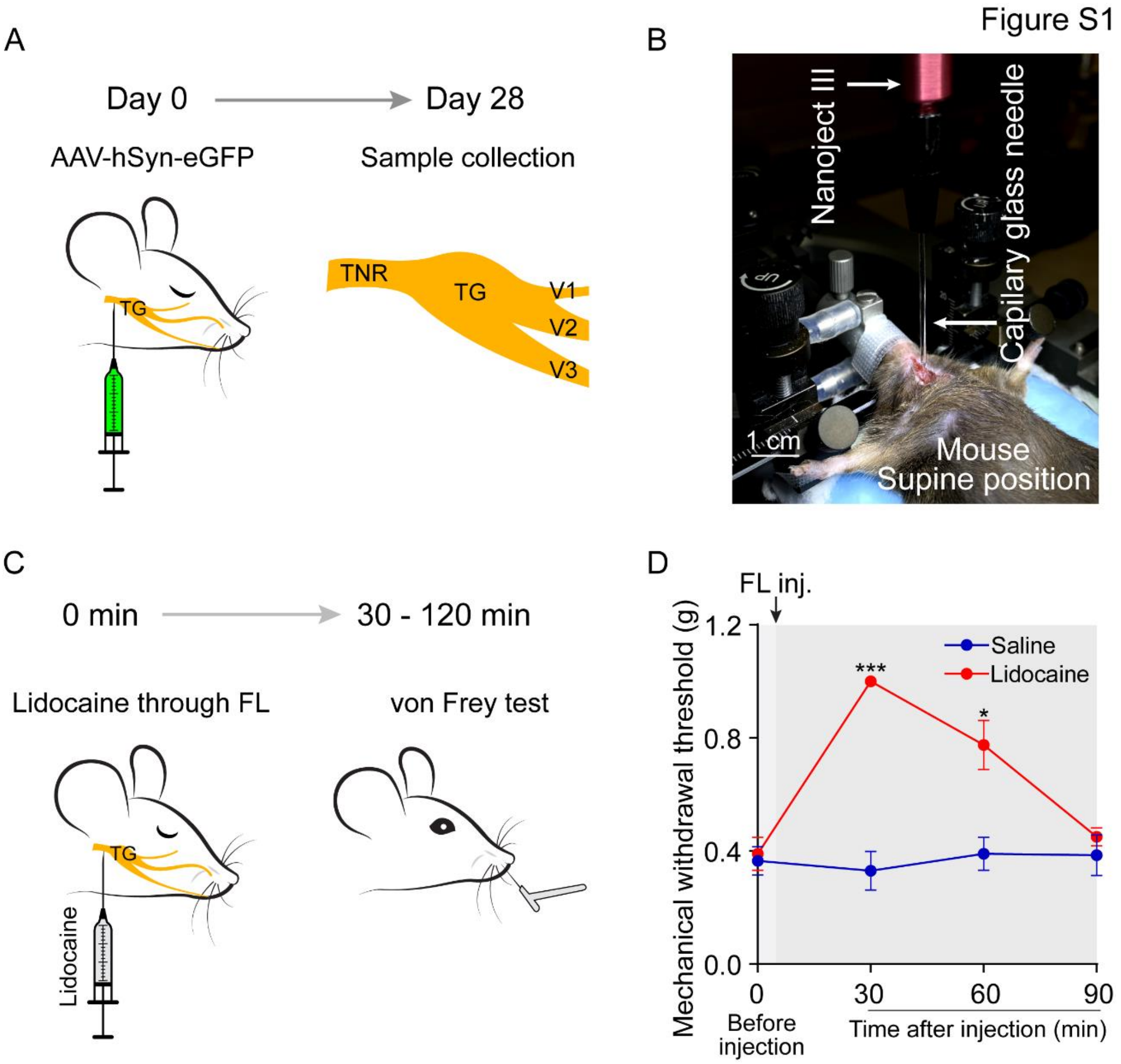

Figure S1. Trigeminal nerve root injection. A-B) AAV injection to the trigeminal nerve root. **A)** Diagram depicting AAV injection to the trigeminal nerve root. **B)** A representative picture of AAV injection. **C-D)** Lidocaine delivery to the trigeminal nerve root increased mechanical withdrawal threshold. **C)** Diagram depicting lidocaine delivery through the foramen lacerum. **D)** Facial mechanical withdrawal threshold. Mice received either saline or lidocaine injection (n=8 per group) through the foramen lacerum. Mechanical withdrawal thresholds were determined before and after injection at indicated time points. Mean ± SEM, there was significant difference between the two groups using two-way ANOVA test. Post-hoc Bonferroni test revealed difference at indicated time points, *$p<0.05$, ***$p<0.001$.

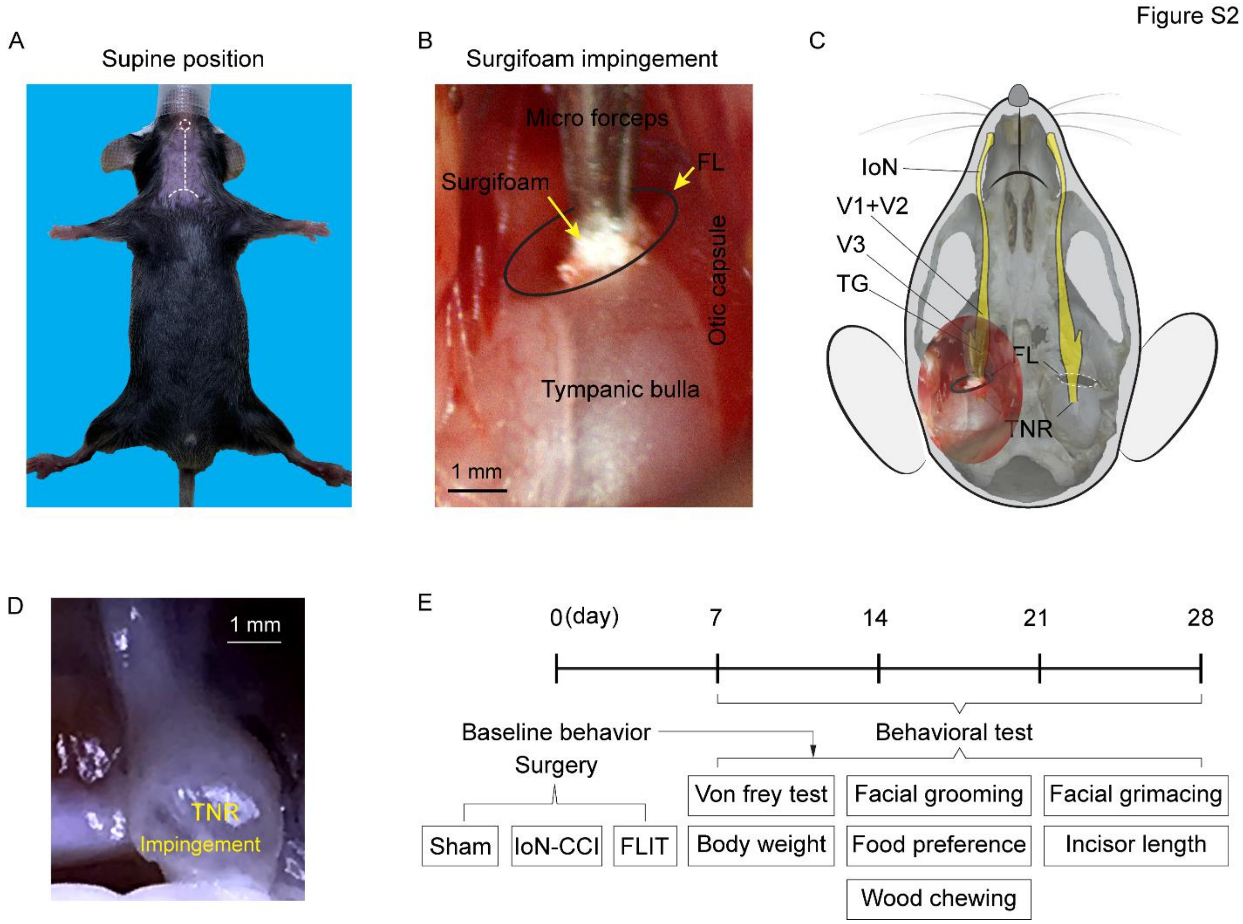

Figure S2. Surgical procedure of FLIT. A) Midline neck incision to access the foramen lacerum. **B)** Surgifoam delivery through the foramen lacerum, picture taken under surgical microscope at 7X-10X magnification. **C)** FLIT procedure picture overlayed in a mouse skull base. **D)** A picture of trigeminal nerve impingement taken at weeks post surgery when mouse brain and dura covering trigeminal nerve were removed. **E**) Timeline of behavioral assessment after the FLIT procedure.

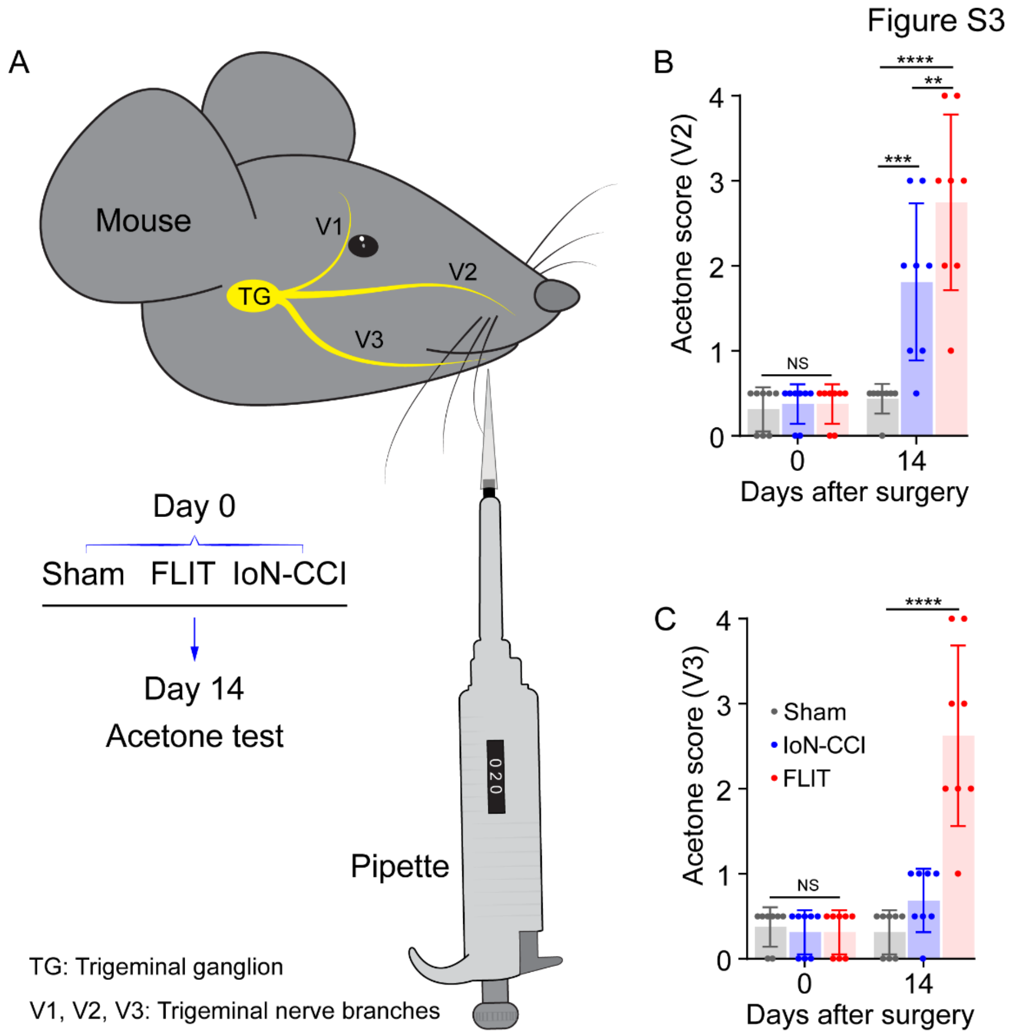

Figure S3: Trigeminal nerve root compression results in cold sensitivity in mice (n=8 per group). Baseline behaviors were tested before surgeries at day 0, acetone test was performed at day 14 after surgeries. **A)** Diagram of experiment. **B)** Acetone scores in the V2 branch of trigeminal nerve; **C)** Acetone scores in the V3 branch of trigeminal nerve branch. Bar chats represent mean ± SD with each dot representing individual animal. One-way ANOVA followed by Tukey post hoc test was performed for comparison among groups. n=8, $**P<0.01$, $***P<0.001$, $****P<0.0001$. NS: not significant ($P>0.05$).

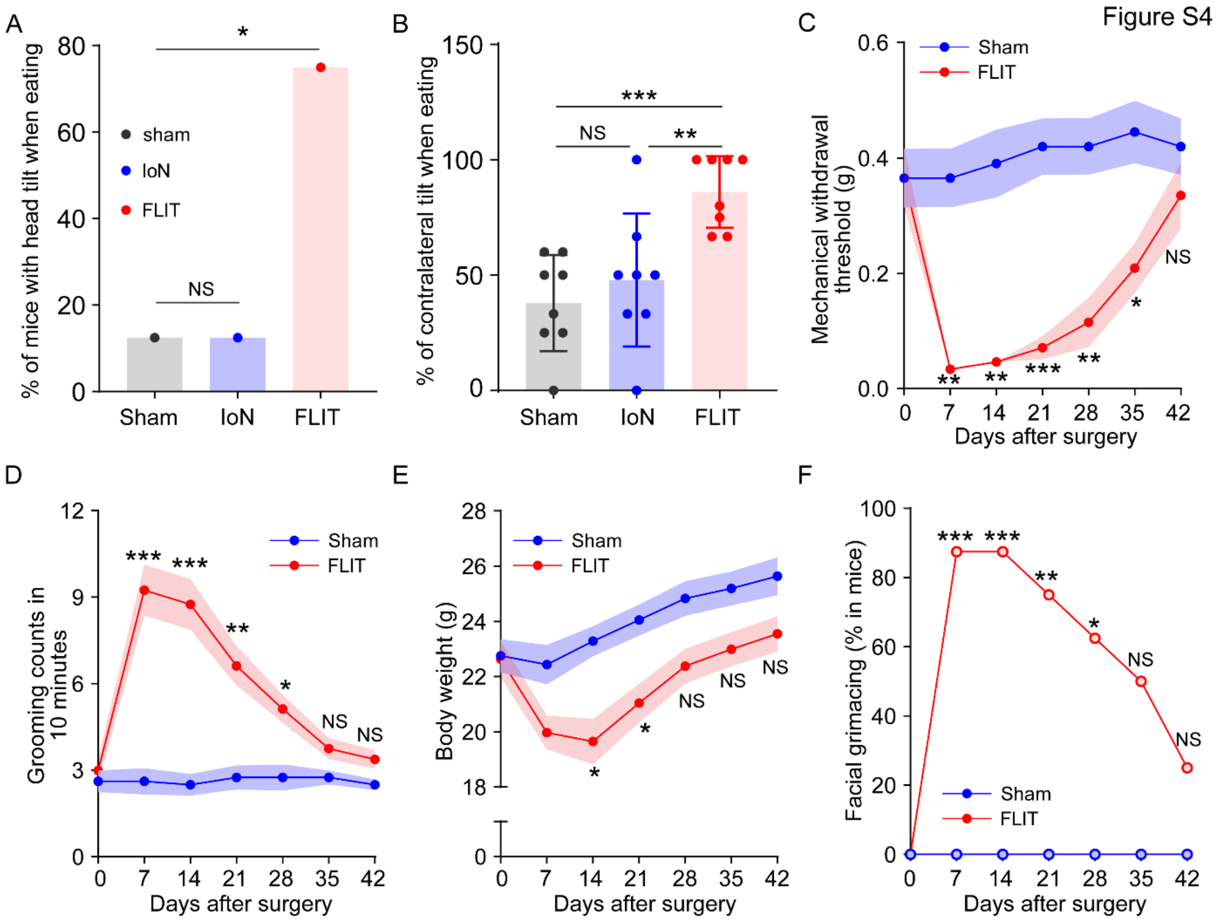

Figure S4. Supplementary behaviors of the FLIT model. **A-B)** FLIT led to preference of contralateral side for eating in mice (n=8 per group). **A**) percent of mice eating with tilt head; **B**) percent of contralateral tilt events when eating, mean ± SD. One-way ANOVA followed by Tukey post hoc test was performed for the comparison among groups. n=8, *P<0.05, **P<0.01, ***P<0.001. NS: not significant. **C-F**) Longitudinal follow up of FLIT mice behavior, n=8 per group, shown line charts represent mean ± SEM except for panel F): **C**) mechanical withdrawal threshold; **D**) grooming counts in 10 minutes; **E**) body weight; **F**) Percentage of mice with facial grimacing. There was significant difference among the two groups using two-way ANOVA test. Post-hoc Bonferroni test revealed difference at indicated time points, FLIT vs. Sham, *p<0.05, **p<0.01, ***p<0.001. Fisher's exact test was used for panel F), *p<0.05, **p<0.01, ***p<0.001, NS: not significant (P>0.05).

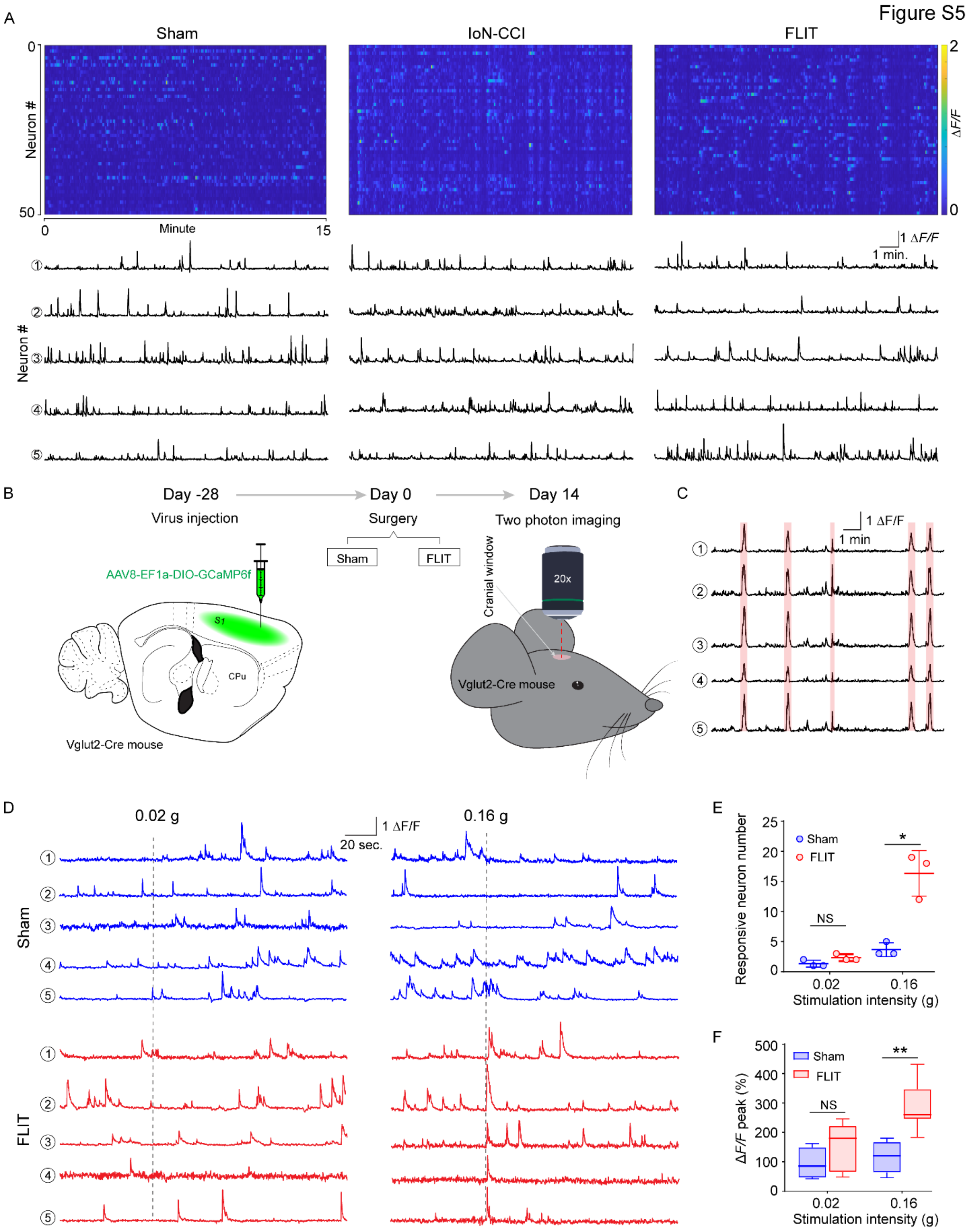
Figure S5
A
Sham
IoN-CCI
FLIT
Neuron #
Minute
0
15
50
2
ΔF/F
1 ΔF/F
1 min.
B
Day -28
Day 0
Day 14
Virus injection
Surgery
Two photon imaging
Sham
FLIT
AAV8-EF1a-DIO-GCaMP6f
S1
CPu
Vglut2-Cre mouse
Cranial window
20x
Vglut2-Cre mouse
C
1 ΔF/F
1 min
D
0.02 g
0.16 g
1 ΔF/F
20 sec.
Sham
FLIT
E
Responsive neuron number
Sham
FLIT
NS
*
0.02
0.16
Stimulation intensity (g)
F
ΔF/F peak (%)
Sham
FLIT
NS
**
0.02
0.16
Stimulation intensity (g)

Figure S5. Supplementary two-photon imaging data. A) Baseline neural activity in the S1 prior to surgery. Mice were injected with AAV8-CaMKII-GCaMP6f virus 28 days prior to imaging. Before FLIT or IoN-CCI surgeries, two-photon calcium imaging were performed to capture baseline neural dynamics in the S1. **A)** Top panels, representative heatmaps of calcium dynamics. Fifty neurons in each mouse were collected over 15 minutes of imaging time. Lower panels showed representative calcium tracing of five individual neurons in the imaging filed. **B-F**) S1 activity assessed in Vglut2-Cre mouse with Cre-dependent GCaMP6f virus. Vglut2-Cre mice were injected with AAV8-EF1a-DIO-GCaMP6f virus 28 days prior to imaging, n=3 per group. **B)** Experiment Design. **C)** Representative neuronal calcium tracing in the FLIT group demonstrating synchronized activities. **D)** Neuronal calcium activities in responses to mechanical stimuli. Shown figures represent three animals per group. **E)** Number of responsive neurons. Mean ± SD. **F)** $\Delta F/F$ peak in responses to mechanical stimuli. Box represents 25th, 50th and 75th percentile and whisker represents minimum/maximum values. Unpaired t test, $*P<0.05$; $**P<0.01$; NS: not significant ($P>0.05$).

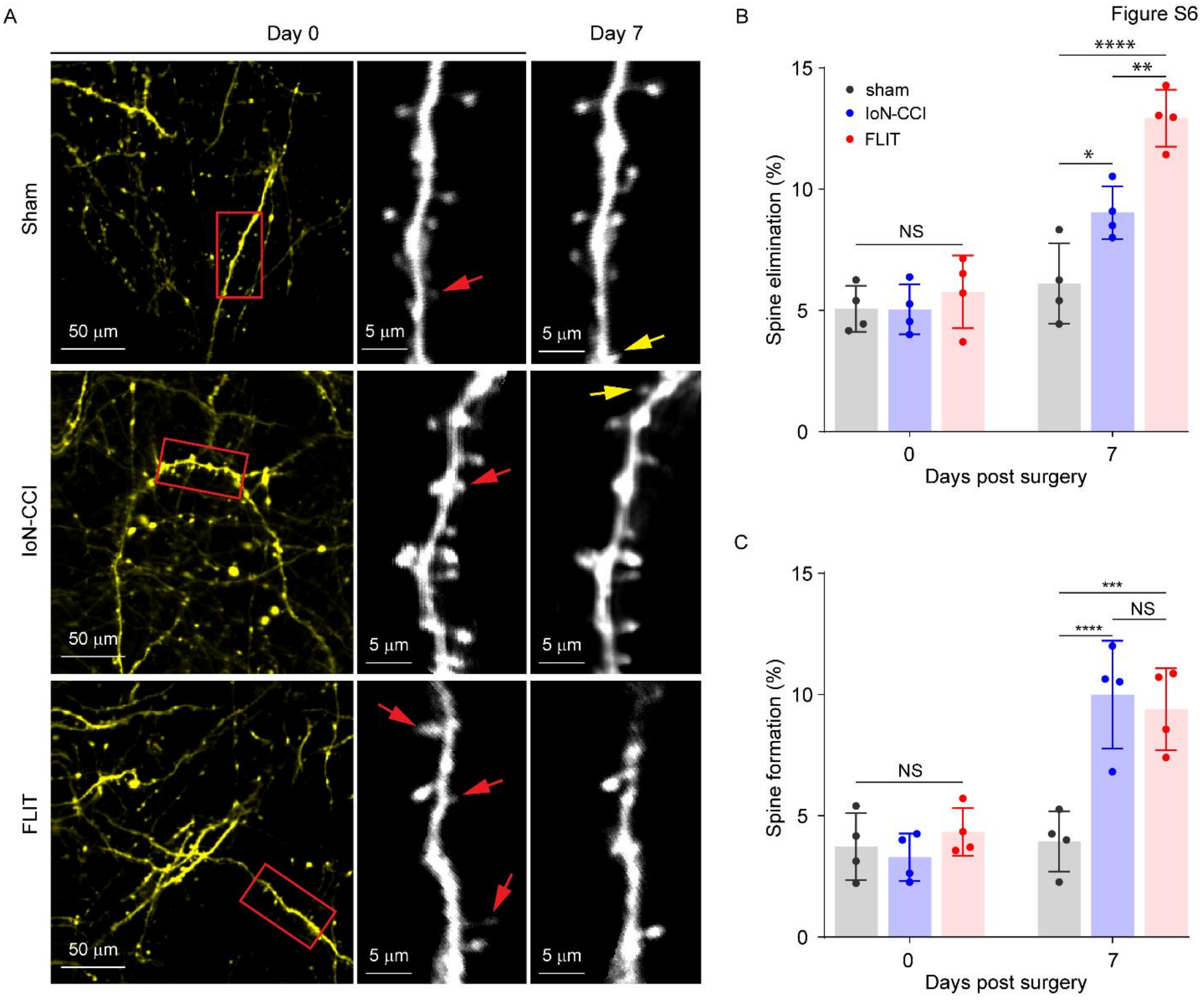

Figure S6. Trigeminal nerve injury leads to dendritic spines turnover. Mice received cranial window implantation 4 weeks prior to baseline imaging, followed by imaging at day 7 after surgery at the same field of view (n=4 each group). **A)** Representative two-photon images of dendritic spines before and after surgery in each group. Red arrow: eliminated spine following surgery; yellow arrow: formed spine following surgery. Boxed regions in A) were quantified before and after surgeries. **B)** Remarkable spines elimination occurred in mice underwent FLIT surgery. **C)** Spines formation occurred in both IoN-CCI and FLIT group after surgeries. Mean ± SD. One-way ANOVA followed by Tukey post hoc test was carried out for the comparison among groups. *P<0.05, **P<0.01, ***<0.001, ****P<0.0001. NS: not significant (P>0.05).
